# The Effect of Helicobacter pylori Density on Serum Vitamin B12 and Folate Levels in Patients With Non-atrophic Gastritis

**DOI:** 10.7759/cureus.45252

**Published:** 2023-09-14

**Authors:** Ibrahim E Pinar, Osman Mavis

**Affiliations:** 1 Department of Internal Medicine, Division of Hematology, Isparta City Hospital, Isparta, TUR; 2 Department of Internal Medicine, Health Sciences University, Istanbul Gaziosmanpasa Training and Research Hospital, Istanbul, TUR

**Keywords:** non-atrophic gastritis, serum vitamin b12, folate deficiency, density, helicobacter pylori, impact

## Abstract

Introduction

Chronic infection with *Helicobacter pylori* (Hp) is an essential cause of gastrointestinal pathologies in adults. Despite being a microorganism proven to play a role in vitamin B12 deficiency by causing gastric atrophy, Hp’s role in patients with non-atrophic gastritis has not been fully explained. Our study investigated whether the presence and density of Hp is related to vitamin B12 and folate deficiencies in patients with non-atrophic gastritis.

Methods

This retrospective cohort study analyzed the following parameters, vitamin B12, folate, and mean red blood cell volume (MCV) in the hemogram; these were measured simultaneously in patients diagnosed with non-atrophic gastritis who had undergone gastroscopy to investigate Hp levels. Patients with conditions that could have caused vitamin B12 and folate deficiencies were excluded from the study. The study included 244 patients who met the criteria. The Sydney classification was used for histopathologic grading and staging of gastric biopsies of patients with gastritis.

Results

There was no relationship between the presence and density of Hp with vitamin B12 levels. However, folate levels were significantly lower in Hp-positive patients than in Hp-negative patients (p = 0.017). Folate levels were substantially lower in patients with chronic pan-mucosal gastritis than in patients with chronic inactive gastritis (p = 0.034). Statistically, a significant difference was found between folate levels on the basis of neutrophil activity and inflammation score (p = 0.011 and p <0.001, respectively).

Conclusions

Although there was no statistically significant relationship between the presence and density of Hp and vitamin B12, our study found an association between folate levels and Hp density. This may be associated with the time for the depletion of vitamin B12 and folate stores and the relatively early stage of gastritis. In cases with vitamin B12 and folate deficiencies, appropriate studies should be performed for specific epidemiological reasons in respective fields.

## Introduction

*Helicobacter pylori* (Hp) is one of the most common pathogens of bacterial infection, and its prevalence is approximately 50% worldwide [1]. Humans constitute the main reservoir for Hp; low socio-economic status is a substantial risk factor for Hp infection [2]. Despite being a non-invasive microorganism, it strongly stimulates inflammation and, in parallel, creates a robust immune response. Bacterial urease activity forms the basis of many non-invasive and invasive tests to diagnose the infection; therefore, it is clinically significant. In addition to causing acute and chronic gastritis, Hp is a microorganism whose role has been proven in duodenal ulcers, gastric adenocarcinoma, and gastric mucosa-associated lymphoid tissue lymphoma [3]. Additionally, it has been suggested that conditions, such as coronary heart disease, iron deficiency anemia, autoimmune thrombocytopenia, diabetes mellitus, and vitamin B12 deficiency, are associated with Hp [4-9]. In a study by Dholakia et al., although Hp infection rates were similar in patients with and without B12 deficiency, patients with B12 deficiency had remarkably less superficial gastritis and substantially more atrophic gastritis compared with non-B12 deficient patients [8]. Still, the relationship between these has not yet been fully elucidated. In patients diagnosed with folate and vitamin B12 deficiencies, the deficiencies must be treated, but an essential aspect of management should be the determination of the underlying cause.

Several problems may lead to a vitamin B12 or folate deficiency. Pernicious anemia, low dietary intake of vitamins (like a vegan diet), gastric surgeries, and intestinal diseases such as celiac or Crohn's disease may cause vitamin B12 deficiency. Pregnant and breastfeeding women have an increased demand for folate. Medications, including anticonvulsants and proton pump inhibitors, and excessive alcohol intake may affect folate absorption into the body [10]. In a prospective cohort study conducted in Turkey, Hp has been observed to be a causal agent in the development of vitamin B12 deficiency, and Hp eradication improved vitamin B12 levels and anemia [11]. A recently published study highlights the role of Hp infection in vitamin B12 deficiency in developing countries [12]. In Hp-negative patients, excessive bacterial growth in the gastrointestinal system may also reduce cobalamin absorption. Therefore, reducing anaerobic load with a broad-spectrum antibiotic regimen may improve food cobalamin malabsorption independent of Hp eradication [13]. On the other hand, in some studies, the presence of Hp has not affected vitamin B12 levels [14].

Although the cause-effect relationship between Hp-induced gastric atrophy and vitamin B12 deficiency is well understood, this relationship is not clear in patients with non-atrophic gastritis. In atrophic gastritis with low gastric acid secretion and possibly insufficient intrinsic factor production, both food-bound and supplemental vitamin B12 are poorly absorbed [10]. Additionally, vitamin B12 deficiency may cause both folate metabolic disorders and exacerbate existing folate deficiency. The effects of Hp infection on vitamin levels are not independent of each other, and there are interactions between many different vitamins [15].

Our study aimed to investigate whether the presence and density of Hp were associated with folate and vitamin B12 deficiencies in non-atrophic gastritis patients, who did not have any diseases or factors that could have affected these levels.

## Materials and methods

Study design

The study was carried out retrospectively on 244 patients aged between 18 and 65 years, who had undergone gastroscopy to investigate levels of Hp. Their folate and vitamin B12 levels were measured simultaneously with the gastroscopy time in Istanbul Gaziosmanpasa Training and Research Hospital between July 2014 and July 2015. Additionally, hemograms were analyzed to determine MCV levels. The cases were classified with respect to the histologic properties and Hp intensity. According to a local laboratory assessment, a vitamin B12 level below 200 pg/mL was defined as vitamin B12 deficiency and a folate level below 2 ng/mL was defined as folate deficiency.

Patients with a history of organic or metabolic diseases, gastrointestinal bleeding, or gastric surgery, pregnant women, vegetarians, alcoholics, and participants who had received vitamin B12 replacement and blood transfusions in the previous six months were excluded from the study. Patients who used proton pump inhibitors, H2 receptor blockers, nonsteroidal anti-inflammatory drugs, metformin, and eradication therapy for Hp infection within the previous six months were also excluded from the study.

Hp densities, neutrophil activities, and inflammation scores were investigated from the biopsy results which were obtained from the gastric antrum of the patients who had undergone gastroscopy. Sydney classification was used for histopathological grading and staging of gastric biopsies of patients with gastritis. Subjects with positive Hp, neutrophil activity, and inflammation scores were subdivided into mild (+), moderate (++), and severe (+++). Hp density, neutrophil activity, and inflammation scores were compared concurrently with the subgroups, categorized with respect to the positivity ratings of positive cases. Neutrophil activation is defined as infiltrating the lamina propria or superficial epithelium and is graded according to severity.

Statistical analysis

The Number Cruncher Statistical System (NCCS; Kaysville, Utah, USA) program was used for statistical analysis. In the study data analysis, descriptive statistics such as mean, standard deviation, median, frequency, ratio, minimum, and maximum were used and expressed with graphical analyses. The expected distributions of the quantitative data were analyzed with the Shapiro-Wilk test. Mann-Whitney U test compared the two groups of quantitative variables without normal distribution. The Kruskal-Wallis test was used for two-to-many comparisons of the non-normal distribution of quantitative variables. The Bonferroni correction for multiple tests was used to adjust for significance values. Spearman correlation analysis was used to evaluate the relationships between quantitative variables. Statistical significance was accepted as p <0.05.

## Results

A total of 244 participants met the eligibility criteria for the study. The median age was 50 (18-65) years; 33.2% of the patients (n = 81) were male, and 66.8% (n = 163) were female.

Hp was positive in 64.3% (n = 157) of the study cohort. The neutrophil activity was positive in 66.8% (n = 163) of the cases, and chronic inflammation was positive in 83.6% (n = 204). The histopathological diagnoses of the patients were chronic inactive gastritis in 27.5% (n = 67), chronic superficial gastritis in 21.3% (n = 52), chronic active gastritis in 41.0% (n = 100), and chronic panmucosal gastritis in 10.2% (n = 25). The distribution of descriptive and clinical characteristics is shown in Table 1.

**Table 1:** Descriptive and clinical features of the study cohort CAG: Chronic active gastritis, CIG: Chronic inactive gastritis, CPG: Chronic panmucosal gastritis, CSG: Chronic superficial gastritis.

Parameters		Median (min-max)	Mean ± SD
Age (years)		50 (18 - 65)	47.9 ± 11.4
Folic acid (ng/mL)		7.6 (3.1 - 18.9)	8 ± 3
MCV (fL)		87.5 (49 - 126)	85.7 ± 8.7
Vitamin B12 (pg/mL)		198 (17 - 924)	238.1 ± 128.9
		n	%
Gender	Male	81	33.2
	Female	163	66.8
Helicobacter pylori	Negative	87	35.7
	Positive	157	64.3
	Mild (+)	67	27.5
	Moderate (++)	55	22.5
	Severe (+++)	35	14.3
Pathologic diagnosis	CIG	67	27.5
	CSG	52	21.3
	CAG	100	41.0
	CPG	25	10.2
Neutrophil activity	Negative	81	33.2
	Mild (+)	78	32.0
	Moderate (++)	63	25.8
	Severe (+++)	22	9.0
Inflammation score	Negative	40	16.4
	Mild (+)	84	34.4
	Moderate (++)	85	34.9
	Severe (+++)	35	14.3

Vitamin B12 results

There was no statistically significant correlation between folate and vitamin B12 levels with patients’ age, sex, and MCV values (Table 2).

**Table 2:** Comparison of vitamin B12 and folic acid levels with age and MCV r: Spearman correlation coefficient

Parameters		Vitamin B12 (pg/mL)				Folic acid (ng/mL)	
		r		p		r	p
Age (years)		0.015		0.819		0.03	0.731
MCV (fL)		-0.096		0.134		-0.115	0.183
Folic acid (ng/mL)		0.048		0.58			

There was no statistically significant difference between Hp-negative and -positive cases regarding vitamin B12 levels. However, it is noteworthy that the minimum vitamin B12 in Hp-negative cases was 116 pg/mL, while the minimum level in Hp-positive patients was 17 pg/mL. In addition, the maximum B12 vitamin level was measured as 924 pg/mL in Hp-negative cases and as 785 pg/mL in Hp-positive patients. There was no statistically significant difference between Hp-negative cases and mild-, moderate- and severe-density of Hp-positive patients in terms of vitamin B12 levels (Table 3). According to pathological diagnoses, neutrophil activities, and inflammation scores, there were no statistically significant differences in vitamin B12 levels.

**Table 3:** Comparison of vitamin B12 and folic acid levels * Statistical significance, ^a^ Mann-Whitney U test, ^b^ Kruskal Wallis test CAG: Chronic active gastritis, CIG: Chronic inactive gastritis, CPG: Chronic panmucosal gastritis, CSG: Chronic superficial gastritis

Parameters			Vitamin B12 (pg/mL)			Folic acid (ng/mL)	
			Median (min-max)		p	Median (min-max)	p
Gender	Male		192 (17 - 924)		^a^0.638	7.3 (3 - 15.4)	^a^0.216
	Female		199 (64 - 609)			7.7 (3.3 - 18.9)	
Helicobacter pylori	Negative		194 (116 - 924)		^a^0.969	8 (3 - 18.9)	^a^0.017*
	Positive		198 (17 - 785)			7 (3.3 - 15.4)	
Helicobacter pylori	Negative		194 (116 - 924)		^b^0.883	8 (3 - 18.9)	^b^0.041*
	Mild (+)		197 (17 - 785)			6.4 (3.5 - 15.4)	
	Moderate (++)		203 (54 - 537)			8.6 (4 - 11.7)	
	Severe (+++)		194 (64 - 602)			5.7 (3.3 - 12.3)	
Pathologic diagnosis	CIG		194 (34 - 924)		^b^0.474	8.4 (3.5 - 18.9)	^b^0.023*
	CSG		204 (64 - 737)			7.8 (3.5 - 15.6)	
	CAG		203 (17 - 785)			7.4 (3.3 - 16)	
	CPG		167 (101 - 602)			6.4 (3.1 - 12.3)	
Neutrophil activity	Negative		188 (34 - 924)		^b^0.403	8.5 (3.1 - 18.9)	^b^0.011*
	Mild (+)		209 (17 - 785)			7.7 (3.5 - 15.6)	
	Moderate (++)		209 (54 - 737)			7.1 (3.3 - 16)	
	Severe (+++)		179.5 (88 - 361)			5.6 (3.5 - 10.6)	
Inflammation score	Negative		202 (109 - 924)		^b^0.653	10.4 (3.5 - 18.9)	^b^<0.001*
	Mild (+)		205 (64 - 609)			8 (3.1 - 15.6)	
	Moderate (++)		197 (17 - 785)			7.1 (3.7 - 16)	
	Severe (+++)		194 (88 - 361)			4.8 (3.3 - 10.6)	

Folate results

In Hp-positive patients, the folate levels were significantly lower compared to Hp-negative patients (p = 0.017 (Table 3).

Although a statistically significant correlation existed between Hp density and folate level (p = 0.041), this could not be confirmed on adjustment of significance values using Bonferroni correction.

**Figure 1:**
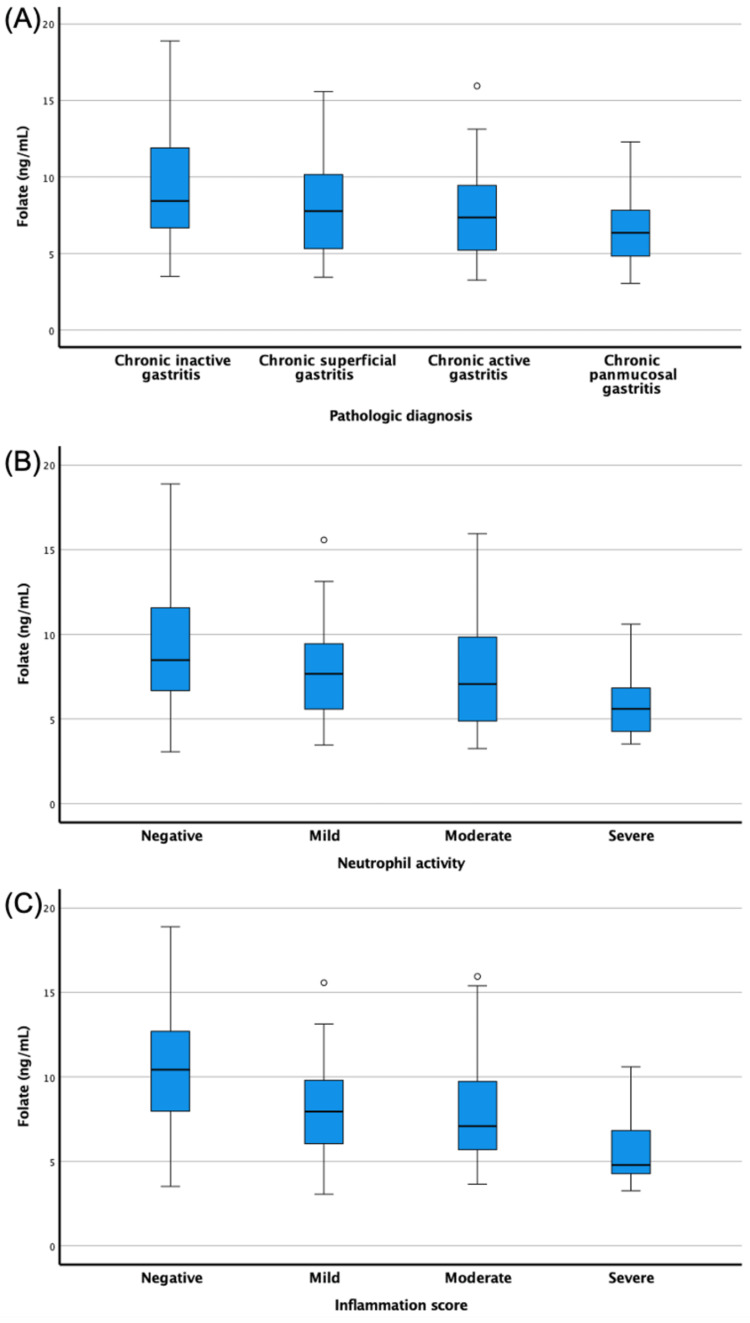
(A) Distribution of folic acid level by pathological diagnosis, (B) Distribution of folic acid level by neutrophil activity, (C) Distribution of folic acid level by inflammation score

A statistically significant difference was found between pathological diagnoses regarding folate levels (p = 0.023) (Figure 1A). Folate levels were significantly lower in chronic panmucosal gastritis than in chronic inactive gastritis (p = 0.034) (Table 4).

**Table 4:** Pairwise comparisons of pathological diagnoses by folate level * Statistical significance, ^a^ the Bonferroni correction for multiple tests has adjusted significance values.

Pathologic diagnosis	p (Adj. Sig.)^a^
Chronic active gastritis vs. chronic superficial gastritis	1
Chronic panmucosal gastritis vs. chronic active gastritis	1
Chronic panmucosal gastritis vs. chronic superficial gastritis	1
Chronic superficial gastritis vs. chronic inactive gastritis	0.459
Chronic active gastritis vs. chronic inactive gastritis	0.143
Chronic panmucosal gastritis vs. chronic inactive gastritis	0.034*

A statistically significant difference was found for neutrophil activity and inflammation score in terms of folate levels (p = 0.011 and p <0.001, respectively) (Figure 1B). In the pairwise comparison of neutrophil activity, folate levels were significantly lower in patients with severe neutrophil activity than in patients with negative neutrophil activity (p = 0.019). In the pairwise comparison of inflammation scores, folate levels were substantially lower in patients with severe inflammation than those with negative and mild inflammation scores (p = 0.027 and p <0.001, respectively). Patients with moderate inflammation scores also had substantially lower folate levels than those with negative scores (p = 0.013) (Table 5 and Figure 1C).

**Table 5:** Pairwise comparisons of neutrophil activity and inflammation score by folate level * Statistical significance, ^a^ The Bonferroni correction for multiple tests has adjusted significance values.

	Neutrophil activity	Inflammation score
	p (Adj. Sig.)^a^	p (Adj. Sig.)^a^
Severe vs. Moderate	0,714	0,161
Severe vs. Mild	0,334	0,027*
Severe vs. Negative	0,019*	<0,001*
Moderate vs. Mild	1	1
Moderate vs. Negative	0,184	0,013*
Mild vs. Negative	0,420	0,128

## Discussion

Many recent studies have evaluated the relationship between Hp infection and micronutrient malnutrition. Hp infection has led researchers to believe that a vitamin B12 deficiency could have been the cause [16-19]. The most common cause of a malabsorption-related vitamin B12 deficiency is the inability to extract cobalamin from food throughout the gastrointestinal tract. If Hp-associated gastritis progresses to gastric atrophy, it might cause malabsorption of vitamin B12 due to hypochlorhydria [20]. In our study, we evaluated patients who did not exhibit gastric atrophy. To the best of our knowledge, this is one of the comprehensive study, assessing the relationship between Hp density with folate and vitamin B12 levels rather than the detection of Hp.

To assess the impact of Hp on serum vitamin B12 and folate levels, all factors, which are affected by a great variety of risks, must be considered. For this purpose, we intended to conduct a literature review concerning all other risk factors for vitamin B12 and folate deficiency, and we also limited the eligibility criteria for participation in the study. This study shows that Hp and its density are not related to vitamin B12 levels in patients with non-atrophic gastritis.

Rasool et al. have demonstrated that Hp infection did not affect folate, vitamin B12, and homocysteine levels in 132 patients with functional dyspepsia. In line with our study, mean vitamin B12 levels in Hp-positive patients did not differ substantially from those in the Hp-negative patient group [14].

Anaya-Loyola et al., in a study conducted on 191 women living in rural areas, have shown that mean serum gastrin, pepsinogen I concentrations, and vitamin B12 levels did not differ between Hp-seropositive and -seronegative patients. Their study supports the thesis that in a rural population, vitamin B12 intake is a stronger predictor in assessing vitamin B12 levels compared to stomach function [21]. Although Hp density in gastric biopsy samples was not examined in their study, our data also revealed that Hp density did not affect vitamin B12 levels in support of their conclusion.

In a cross-sectional study by Stettin et al., conducted on 90 patients, mean CRP concentration was substantially higher in the Hp-positive subjects compared to the controls. However, B12 methylmalonic acid mean concentrations were not substantially different between the patient groups. However, unlike our study, mean homocysteine and folic acid concentrations were almost identical in both patient groups [22].

On the other hand, contrary to our data, although a significant relationship has been found in some studies between Hp and vitamin B12 [23-25], no association with folate levels has been found [23]. Several, but not all, studies supporting the association between B12 deficiency and Hp infection have described whether patients with atrophic gastritis are included. In a paper by Ulasoglu et al., a low B12 level was substantially correlated with atrophy [25]. In a study by Kalkan et al., in patients >60 years of age diagnosed with non-atrophic gastritis, Hp infection, neutrophil activation, and inflammation were significantly more common in those with B12 deficiency [26]. A considerable challenge is correlating the presence and density of Hp with vitamin B12 levels. Our study pointed to Hp infection in many patients with negative or low neutrophil activity, absence of active gastritis, and lack of marked increases in inflammatory markers. Evidence of causality between Hp and vitamin deficiencies has mixed results; this may often be explained by how long the infection has been present or that different genotypes of bacteria have other effects. At the time of Hp infection diagnosis, some patients may have been dealing with the disease for a long time. In contrast, others may have acquired the condition relatively recently.

Kaptan et al. reported that Hp eradication improved vitamin B12 deficiency in adults with low serum vitamin B12 levels (mean ± standard deviation: 63 ± 30). This suggests that Hp infection is a causative agent for vitamin B12 deficiency in adults [11]. The inconsistency of these findings with our outcomes could be caused by higher vitamin B12 levels in our study group.

In our study, we excluded patients with gastric atrophy; their cause-effect relationship with vitamin B12 deficiency is well known. Our patients consisted only of cases of chronic gastritis, a relatively early stage of gastric inflammation caused by Hp. This may be one of the reasons for our failure to discover an association between Hp infection and vitamin B12.

Several research, but not all, have manifested that the presence of Hp infection has a reverse relationship with serum levels of folate [25]. The negative effect of Hp infection on the decrease in folate absorption can be explained by the increase in pH or decrease in vitamin C concentration in gastric juice; a condition frequently found in Hp-positive cases [27]. On the other hand, one of the reasons for our discovery of a substantial relationship between folate deficiency and Hp infection could be the faster depletion of folate stores than vitamin B12, and thereby earlier onset of folate deficiency.

Herbert defined the sequential stages of vitamin B12 deficiency development and the hematological and biochemical changes as a negative vitamin B12 balance progressed. Total plasma vitamin B12 concentration is not a highly sensitive or specific marker for a reliable diagnosis of vitamin B12 deficiency. Compared to total plasma vitamin B12 levels, serum holotranscobalamin is an earlier and more sensitive indicator of vitamin B12 deficiency [28]. This is based on the properties of holotranscobalamin, which constitutes a small percentage of total vitamin B12 and has a short half-life. On the other hand, holohaptocorrin has a much longer half-life compared to holotranscobalamin in serum, and there is a large store of vitamin B12 in the liver. Therefore, holohaptocorrin falls much more slowly compared to holotranscobalamin. The holotranscobalamin immunoassay, commercially available for the diagnosis of vitamin B12 deficiency, is presented as a solution against the unmet need for the early detection of negative vitamin B12 balance [29]. The use of holotranscobalamin immunoassay for detecting vitamin B12 deficiency is critical and will shed light on similar future research on the subject.

In our study, we only measured the total serum vitamin B12 levels. Our results, based on serum vitamin B12 levels, likely indicate the presence of both non-bioavailable and bioavailable cobalamin. One of the limitations of our study is that we did not use markers such as plasma homocysteine or holotranscobalamin as an earlier and more sensitive marker in the evaluation of vitamin B12 deficiency. We are acutely aware of the substantial limitations of our study, in which we are more likely to miss true positive results by measurement of total vitamin B12. The study’s retrospective design and the relatively small number of examined participants may contribute to a joint multicenter and multi-regional collaboration to expand the sample size and therefore improve sample representation in future studies, providing more reliable results. Because of these limitations, relationships should be interpreted cautiously.

## Conclusions

In our study, although folate levels were correlated with Hp concentration, there was no statistically significant difference between the presence and density of Hp with vitamin B12 levels. It can be associated with the depletion time of vitamin B12 and folate stores and the relatively early stage of gastritis. It should be emphasized that Hp is not the only agent of vitamin B12 deficiency. In cases of vitamin B12 deficiency, appropriate tests should be performed for specific epidemiological reasons in each field. Re-evaluation of Hp infection and its relationship with vitamin B12 and folate levels, when subjected to more detailed parameters and large-scale studies, will constitute an essential reference value in treatment planning.
